# The New Mental Board of Control

**Published:** 1913-11-08

**Authors:** 


### The Hospital

The Workers' Newspaper of Administrative Medicine and Institutional Life, Administration, National Insurance and Health.

No. 1428, Vol. LV. SATURDAY, NOVEMBER 8, 1913. PRICE ONE PENNY.

### THE NEW MENTAL BOARD OF CONTROL.

After various misadventures and rejections the Mental Deficiency Bill was passed through Parliament at last in August of this year, and presently will be in full working order. Already, as is noted in another column, the Chairman and two other paid members of the Board of Control set up by the Act have been appointed. The Lunacy Commissioners have seats on this Board *ex officio*, and it only remains to appoint four unpaid members to complete the *personnel* of the new authority. The Chairman of the Board is Sir William Byrne, K.C.V.O., C.B., who is an Assistant Under-Secretary of State for the Home Department, and was Chairman of the Royal Commission on the Feeble-Minded in 1904. And the other two new appointments are equally satisfactory.

What, then, are the functions of this new Board of Control under the Act? For an answer to this question the Act itself must be consulted, since its operation has not as yet been reduced into a settled system. Broadly speaking, the Act charges the Commissioners with the general superintendence of all matters relating to the supervision, protection, and control of the mentally defective, subject to the prior and overriding jurisdiction of already constituted authorities with respect to lunatics. They are to be a body corporate, having the name of "the Board of Control," with a common seal and with power to hold land without licence in mortmain for the purposes of their powers and duties. They may, subject to the approval of the Home Secretary, appoint an administrative committee, to which may be entrusted certain administrative powers and duties catalogued in a Schedule attached to the Act. The duties and powers thus scheduled are exceedingly wide, and a hotchpot clause in the Schedule allows the Home Secretary to extend them to any degree he thinks fit. The Chairman may receive a salary not exceeding £1,800 per annum, and the paid members (including the Lunacy Commissioners) not exceeding £1,500 each; but these are maximum rates, and it is within the competence of the Home Secretary and the Treasury to pay lower salaries to begin with. The present Lunacy Commissioners who join the Board are in any case entitled to enjoy their former emoluments, notwithstanding anything to the contrary in the provisions of the Mental Deficiency Act. It does not transpire whether the Home Secretary has exercised the discretion thus given to him under the Act.

The Board are to be assisted in the performance of their duties by a secretary and by such inspectors and other officers and servants as the Home Secretary and the Treasury may allow. It is specifically enacted that women as well as men are to be thus employed, just as the Board itself must contain at least one female paid and one female unpaid Commissioner. The chief functions of the Board will no doubt be of the nature of supervision over the administration by local authorities of their powers and duties under the Act. But besides this, and the powers of inspection, visitation, and certification which are entrusted to them for the purpose, the Board are also empowered themselves to provide and maintain institutions for defectives of violent or dangerous propensities, and to take any necessary steps for ensuring suitable treatment of the mentally deficient.

In turn the local authorities, who will work under the Board of Control, are charged with important functions in their own areas. The County and Borough Councils are the local authorities specified, and they must constitute a committee for the purposes of the Act, a minority of whom need not be members of the Council at all. These committees must run to earth the mentally defective who are subject to the Act in their respective districts, provide suitable accommodation for them, supervise and protect them, and may even bury them when they die, if thought fit. Furthermore, to assist in the discovery of the feeble-minded by the local committees, it is made part of the duty of local education authorities to report the names and addresses of defective children who are incapable of receiving benefit from instruction in special schools or classes.

It will be observed that the Board of Control have wide powers, but that the Home Secretary, who chooses them and fixes their salaries, is more or less responsible to Parliament through the annual estimates of his Department for what they may do. This should surely serve to placate in some measure the hostility of the small but energetic section which fought so strenuously against the Act in the House of Commons. The general tendency of recent legislation to delegate vaguely limited and enormous powers to bureaucratic bodies has certain disadvantages which have been well illustrated of late years. But it has compensating benefits in that it eliminates very largely the elements of sentimentality and of amateurishness in favour of the trained and level-headed expert in the administration of complex machinery. The Board of Control as now constituted is so strong that, in a few years, given that sensible outlook that is to be expected of its members, it will be practically autocratic, to the vast benefit of future social work in protecting the feeble-minded.

